# Scalable production of biliverdin IXα by *Escherichia coli*

**DOI:** 10.1186/1472-6750-12-89

**Published:** 2012-11-23

**Authors:** Dong Chen, Jason D Brown, Yukie Kawasaki, Jerry Bommer, Jon Y Takemoto

**Affiliations:** 1 Synthetic Bioproducts Center, 620 North 600 East, Utah State University, North Logan, Utah, 84341, USA; 2 Department of Biology, 5305 Old Main Hill, Utah State University, Logan, Utah, 84322, USA; 3 Frontier Scientific, Inc, 195 South 700 West, Logan, Utah, 84323, USA

**Keywords:** Biliverdin IXα, Heme oxygenase, *Escherichia coli*, HO1, Bilirubin, Anti-inflammatory, Biliverdin reductase, Bioreactor

## Abstract

**Background:**

Biliverdin IXα is produced when heme undergoes reductive ring cleavage at the α-methene bridge catalyzed by heme oxygenase. It is subsequently reduced by biliverdin reductase to bilirubin IXα which is a potent endogenous antioxidant. Biliverdin IXα, through interaction with biliverdin reductase, also initiates signaling pathways leading to anti-inflammatory responses and suppression of cellular pro-inflammatory events. The use of biliverdin IXα as a cytoprotective therapeutic has been suggested, but its clinical development and use is currently limited by insufficient quantity, uncertain purity, and derivation from mammalian materials. To address these limitations, methods to produce, recover and purify biliverdin IXα from bacterial cultures of *Escherichia coli* were investigated and developed.

**Results:**

Recombinant *E. coli* strains BL21(HO1) and BL21(mHO1) expressing cyanobacterial heme oxygenase gene *ho1* and a sequence modified version (*mho1*) optimized for *E. coli* expression, respectively, were constructed and shown to produce biliverdin IXα in batch and fed-batch bioreactor cultures. Strain BL21(mHO1) produced roughly twice the amount of biliverdin IXα than did strain BL21(HO1). Lactose either alone or in combination with glycerol supported consistent biliverdin IXα production by strain BL21(mHO1) (up to an average of 23. 5mg L^-1^ culture) in fed-batch mode and production by strain BL21 (HO1) in batch-mode was scalable to 100L bioreactor culture volumes. Synthesis of the modified *ho1* gene protein product was determined, and identity of the enzyme reaction product as biliverdin IXα was confirmed by spectroscopic and chromatographic analyses and its ability to serve as a substrate for human biliverdin reductase A.

**Conclusions:**

Methods for the scalable production, recovery, and purification of biliverdin IXα by *E. coli* were developed based on expression of a cyanobacterial *ho1* gene. The purity of the produced biliverdin IXα and its ability to serve as substrate for human biliverdin reductase A suggest its potential as a clinically useful therapeutic.

## Background

Biliverdin is a linear tetrapyrrole produced by ring cleavage of heme catalyzed by the enzyme heme oxygenase (HO) (E.C.C.1.14.99.3) [1]. In animals, heme cleavage by HO occurs selectively at the α−methene bridge to generate the most physiologically relevant biliverdin IXα isomer. Hence, the term “biliverdin” typically refers specifically to biliverdin IXα, and this usage is applied throughout in this paper. Biliverdin is best known as a degradative intermediate associated with erythrocyte and hemoglobin turnover. It is subsequently reduced via NADPH biliverdin reductase (E.C.C. 1.3.1.24) to bilirubin IXα that in turn is consecutively bound to serum albumin and glucuronic acid for excretion in bile. The overall process serves to eliminate heme - which is toxic when accumulated.

Bilirubin IXα is also known to associate with cell membranes where it quenches the propagation of reactive oxygen species (ROS) [2,3] conferring protection to membrane lipids and proteins against oxidative damage. Thus, an additional function of biliverdin is to serve as the immediate source of bilirubin IXα that in turn acts as a cytoprotective antioxidant. It is not clear if biliverdin is oxidatively regenerated after bilirubin IXα reacts with ROS [4-7]. Though bilirubin IXα is an effective ROS quencher, biliverdin administered at tissue injury/inflammatory sites appears as effective a cytoprotectant as bilirubin IXα [8-13]. Biliverdin‘s effectiveness has been attributed to its hydrophilicity and efficient conversion to bilirubin IXα [1]. In addition, biliverdin interaction with biliverdin reductase signals the downstream production of anti-inflammatory cytokine interferon-10 [14] and the nitrosylation-dependent inhibition of pro-inflammatory TLR4 expression [15]. Thus, biliverdin, acting together with biliverdin reductase, is increasingly recognized as a potential anti-inflammatory therapeutic agent [3,16-18]. Examples of its cytoprotective effects in animal models include those for ischemia/reperfusion following liver [19] and small bowel [10] transplants, vascular injury [20], endotoxic shock [21], vascular intimal hyperplasia [9], and nephropathy [8]. In addition, biliverdin has been reported to inhibit in vitro replication of hepatitis C [22] and other viruses [23,24] and to reverse parameters of type 2 diabetes in mice [25]. The growing list of potential clinical applications for biliverdin suggests a future need for high-quality preparations in ample quantity.

Biliverdin is also produced by microbes and plants [26-30]. In cyanobacteria, red algae, and plants, it serves primarily (and perhaps solely) as precursor to photosensitive linear tetrapyrroles such as phycocyanobilin and phycoerythrobilin [31]. These in turn serve as chromophores for cyanobacterial and red algal light-harvesting phycobiliprotein complexes and the light-sensing receptor phytochrome [27,32]. In these organisms, biliverdin IXα is the predominant isomer produced via HO enzymes with sequence homologies to mammalian HO1 [28,33,34].

To meet the projected pharmaceutical demand for biliverdin, high yield and low cost methods that provide the IXα isomer in high purity and preferably from non-mammalian sources are needed. Currently, commercial biliverdin is predominantly derived by chemical oxidation of bilirubin [35]. The source bilirubin (that occurs in conjugated form) is extracted from mammalian bile under acidic conditions that generate isomers (e.g. IIIα and XIIIα isoforms) and consequently lead to biliverdin preparations of unsuitable purity (e.g. as low as 38% biliverdin IXα [36]). Reported non-mammalian synthesis of biliverdin include *Escherichia coli* cultures expressing HO1 from rat [37,38] and cyanobacteria [39] and yeast cultures supplemented with hemoglobin [40]. In these reports, the amounts of biliverdin produced are not documented or appear low. Biliverdin extracted from salmon bile is reported [41], but the potential for scalable production is not discussed.

Here, we report the use of *E. coli* to synthesize biliverdin and describe procedures for the scalable production of the IXα isomer. This was achieved by sequence optimization of the cyanobacterial *ho1* gene for enhanced expression in *E. coli* and development of growth culture parameters that promote biliverdin production.

## Methods

### *E. coli* strains and vectors

One Shot® TOP10 Chemically Competent *E. coli* (Life Technologies, Carlsbad, CA, USA) was used to construct the recombinant plasmids. BL21 Star™ (DE3) Chemically Competent *E. coli* (Life Technologies, Carlsbad, CA, USA) was used for transformation and protein expression. Expression vector constructions were done with pET101/D-TOPO® (Life Technologies, Carlsbad, CA, USA) and pJexpress 401 (DNA2.0, Menlo Park, CA, USA).

### Construction of expression vectors

#### *pET101-HO*

**Figure 1:**
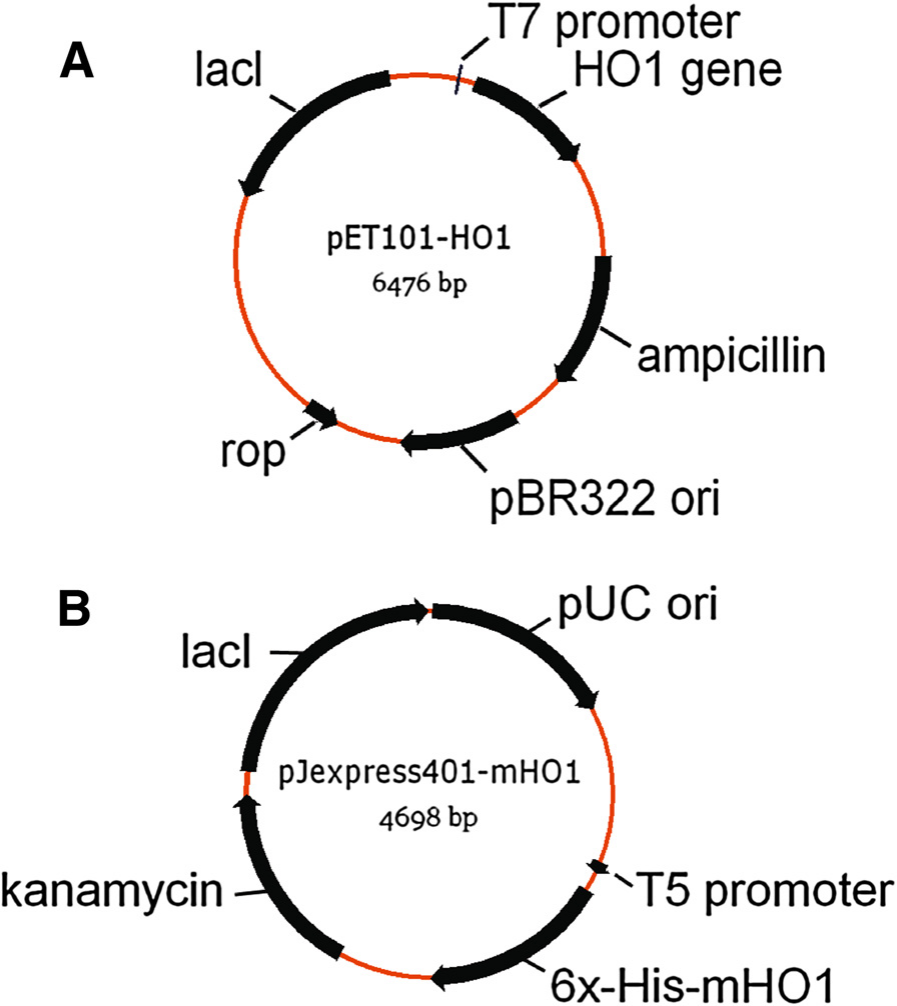
**Gene maps of expression vectors pET101-HO1 (A) and pJexpress401-mHO1 (B). ***ho1* gene expression in (**A**) is controlled by T7lac promoter which consists of a strong bacteriophage T7 promoter and a downstream 25 bp *lac* operator in pET101. For mho1 expression (**B**), an “IP-free” T5 promoter sequence was used with the lac operator placed downstream of the T5 promoter in the vector pJexpress 401.

The heme oxygenase gene (*ho1)* of *Synechocystis* PCC6803 was amplified by PCR of Biobrick gene part BBaI15008 (Registry of Standard Parts, The BioBricks Foundation, http://biobricks.org/) using forward primer 5’-CACC ATGAGTGTC AACTTAGCTTC-3’ and reverse primer 5’-CTAGCCTTCGGAGGTGGCGA-3’ and cloned into pET101/D-TOPO® to generate plasmid vector pET101-HO1 (Figure 1A) with expression under T7lac promoter control according to instructions provided by Life Technologies (Carlsbad, CA) (TOPO® Cloning Reaction Method). The *ho1* gene sequence was verified by DNA sequencing. The vector pET101-HO1 was transformed into BL21 Star™ (DE3) Chemically Competent *E. coli* to give *E. coli* strain BL21(HO1).

#### *pJexpress401-mHO1*

**Figure 2:**
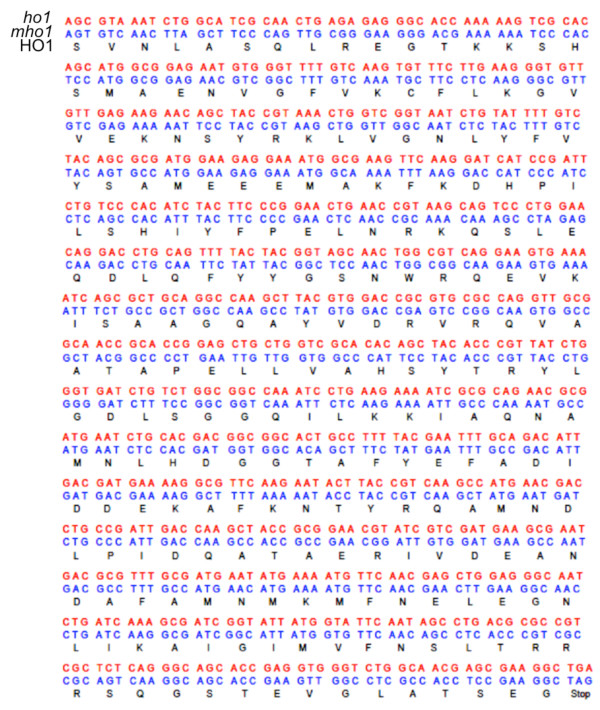
**Gene sequence of *ho1 *of cyanobacterium *Synechocystis *PCC6803 (red), *E. coli *expression optimized *mho1 *gene sequence (blue) and the corresponding translated ho1 protein sequence (black).**

The *ho1* gene sequence was codon optimized for expression in *E. coli* using DNA2.0 Algorithms (DNA2.0, Inc., Menlo Park, CA, USA) (Figure 2). The coding sequence for hexahistidine was incorporated at the 5’ end to provide a 6X His tag at the N-terminus of the synthesized protein. The *E.coli* codon optimized gene (*mho1*) was synthesized and inserted into plasmid vector pJexpress401 by DNA2.0 Inc. (Menlo Park, CA, USA). The resulting vector, pJexpress401-mHO1 (Figure 1B), was transformed into BL21 Star™ (DE3) Chemically Competent *E. coli* to give *E. coli* strain BL21(mHO1).

### Testing carbon sources for biliverdin production

Several carbon sources at different concentrations and in combination were examined for capabilities to support growth and biliverdin synthesis by *E. coli* strains BL21(HO1) and BL21(mHO1). Cultures were grown in 125mL capacity Erlenmeyer flasks on a New Brunswick G76 rotary incubator shaker (30°C, 200 rpm) in 50mL Luria-Bertani (LB) medium [42] with various single carbon sources that included sucrose (1% wt vol^-1^), mannitol (0.1, 1, 2, 5, 10 and 20% wt vol^-1^), sorbitol (1, 5,10 and 20% wt vol^-1^), lactose (1, 2.5, 5 and 10% wt vol^-1^), succinate (2% (wt vol^-1^), malate (2%) or combinations of carbon sources that included mannitol (1% wt vol^-1)^ + glucose (1% wt vol^-1^), sucrose (1% wt vol^-1^) + glucose (1% wt vol^-1^), mannitol (1% wt vol^-1^) + sorbitol (2.5% wt vol^-1^), or mannitol (5% wt vol^-1^) + sorbitol (5% wt vol^-1^). Ampicillin or kanamycin (100μg mL^-1^) was used for selection, and isopropyl-β−thiogalactopyranoside (IPTG) (0.5mM) was added (at cell density with absorbance (1 cm) (A_600_) of ~0.5) as inducer except when lactose was the carbon source. Growth was monitored at A_600_ and the culture color was recorded when stationary phase growth was achieved (24 to 48h). Biliverdin levels were estimated by absorbance spectroscopy using a mM extinction coefficient of 25 at 650nm (1cm light path length) using a SpectraMax Plus384 Absorbance Microplate Reader (Molecular Devices, Sunnyvale, CA, USA).

### Biliverdin production using bioreactor batch cultures

For bioreactor inocula, *E. coli* strains BL21(HO1) and BL21(mHO1) were grown in 50mL of LB medium plus 100μg mL^-1^ ampicillin or kanamycin, respectively, in 250mL capacity Erlenmeyer flasks with rotary shaking (225 rpm) at 37^o^ C to an A_600_ of 2 to 6 with LB medium as a blank control. Inoculum cultures (80mL) were added to 2L of modified New Brunswick Scientific (NBS) medium [43] with 2% (wt vol^-1^) lactose in place of glucose or modified ZY medium [44] composed of per L: 2g lactose, 2.2g glucose, 16g glycerol, 15g N-Z-Amine™ A or 10g Hy Express System II (Sheffield™ Bio-Science, Norwich, NY), 10g yeast extract (FisherScientific), 1mL 2M MgSO_4_, 50mL of 20X NP solution (66g (NH_4_)_2_SO_4_, 136g KH_2_PO_4_, 142g Na_2_HPO_4_ in 1L twice-distilled H_2_O), and 1mL 1000X trace elements solution (50 mL of 1% HCl, 0.675g FeCl_3_, 0.15g CaCl_2_, 0.1g MnCl_2_, 0.015g ZnSO_4_, 0.023g CoCl_2_, 0.015g CuCl_2_, 0.023g NiCl_2_, 0.025g Na_2_MoO_4_, 0.007g H_3_BO_3_ in distilled water to a final volume of 250mL). Separately, 20X NP solution and 2M MgSO4 were autoclaved and 1000X trace elements solution was filter-sterilized, and the solutions were added to complete the preparation of modified ZY growth medium. Batch culture growth was conducted in a New Brunswick Scientific (Endfield, CT, USA) Bioflo 310 Controller bioreactor using BioCommand software with a 5L capacity vessel. A dissolved O_2_ level of 40% was cascade controlled and monitored by gassing with O_2_ (0 - 50%) and air (0.75 - 4 SLPM). No antifoam was used. For fed-batch experiments, a 200mL solution of 10% (wt vol^-1^) glycerol, 2% (wt vol^-1^) lactose, and with or without 5% (wt vol-1) peptone was continuously fed (8mL h^-1^ L^-1^) during exponential growth beginning 4h after inoculation. Cell culture absorbance (A_600_) was approximately 10 at 4h and 29 at 11h after culture inoculation. Growth was terminated approximately 25h after inoculation. Green material (containing biliverdin) accumulated in foam above the culture liquid surface and on the inner surfaces of the bioreactor vessel and in the foam over-flow material that was siphoned into a flask outside the vessel. The pigmented material was collected using methanol or distilled water as necessary and the pH of the final suspension was lowered to 4.3 or 4.5, respectively, to promote biliverdin precipitation. The recovered material was centrifuged at 7477xg for 6 min, and the sedimented blue-green material was suspended in methanol. Non-sedimenting biliverdin in aqueous fractions was recovered by readjusting the pH to 4.3 followed by re-centrifugaton and suspension of the green pellet in methanol. The pooled methanolic solutions were placed on a rotating shaker (225 rpm) at room temperature for 15min. The solution was centrifuged at 4500xg for 4min to remove particles from solution. Fresh methanol was added to the pellet, and the extraction repeated. The extraction is further repeated with distilled methanol until the A_650_ of a 1:10 dilution of the supernatant fluid is less than 0.5. The amounts of biliverdin recovered were quantitated by HPLC with comparisons to known amounts of authentic biliverdin IXα (Frontier Scientific, Inc., Logan, Utah).

Larger (100L) batch cultures of *E. coli* strain BL21(HO1) were grown at 37°C with NBS medium containing 2% (wt vol^-1^) lactose in a B. Braun UE-100D bioreactor (B. Braun Melsungen AG, Germany). Fed-batch mode was not used. *E. coli* strain BL21(HO1) inoculum cultures (4L) were grown overnight at 37°C in LB medium in Bioflo310 bioreactors. Inoculum cultures (4L) were added to 100L growth medium and growth was terminated 24h following inoculation. Biliverdin was collected, extracted and purified as described above for the 2L bioreactor batch cultures.

### HO identification and activity

#### HO cell extraction

Aliquots (48 to 400mL) of bioreactor batch cultures of *E. coli* strain BL21(mHO1) were collected at 2, 5, 10, 15 and 2h after inoculation, centrifuged (4500xg, 5min), and the supernatant liquid discarded. The sedimented cell pellets were stored at −20°C. The cells were extracted, and proteins were recovered from Ni-NTA columns using the QIAexpress® Ni-NTA fast Start Kit (QIAGEN, Valencia, CA, USA) according to procedures described in the kit manual.

#### SDS-PAGE

Twenty μL of each protein solution from Ni-NTA column purification were added to 20μLof SDS-PAGE sample buffer (Bio-Rad, Hercules, CA, USA), heated for 10min with boiling water, and centrifuged briefly. Supernatant liquid aliquots (30μL) were loaded into wells of Bio-Rad Criterion Precast Gels and electrophoresed in a Bio-Rad Criterion precast Gel System. The gel was stained using Bio-safe™ Coomassie G-250 (Bio-Rad, Hercules, CA, USA). Precision Plus Protein Prestained Standards (Bio-Rad Laboratories, Hercules, CA, USA) was used for estimation of protein molecular size.

#### Identification

Ni-NTA column purified protein samples were reduced and alkylated with iodoacetamide. The resulting peptides were concentrated on a ZipTip micropurification column and eluted onto an anchorchip target for analysis on a Bruker Autoflex III MALDI TOF/TOF instrument (performed by Alphalyse, Inc., Palo Alto, CA, 94306). The peptide mixture was analyzed in positive reflector mode for accurate peptide mass determination. MALDI MS/MS analyses were performed on 8 separate peptides for partial peptide sequencing. The MS and MS/MS spectra were combined and analyzed using Mascot software and NCBI protein databases.

#### HO activity

Harvested *E. coli* strains BL21(mHO1) and BL21 Star™ (DE3) cells were washed and suspended in assay buffer (50 mM Tris–HCl, pH 7.7, 10% wt vol^-1^ glycerol) and 1mM EDTA, and disrupted three times using a French press cell operated at 18,000 psi. The lysate was centrifuged at 15,000x*g*, and the supernatant fraction was used for HO activity assays similar to published procedures [28,45]. The enzyme reaction mixture (500μL) contained assay buffer, 40μM methemalbumin, 2.5mM Tiron, 20μg mL^-1^ ferredoxin, 0.02 units of ferredoxin reductase (Sigma-Aldrich, St. Louis, USA), and cell lysate (0.128mg protein). The reaction was initiated with the addition of 0.2mg of NADPH and the mixture was incubated at 37°C for 20min in the dark. The mixture was then extracted and esterified [46] and biliverdin dimethyl ester was quantitated by HPLC using a Beckman C18 Ultrasphere column (4.6 mm x 15 cm), elution with methanol, and absorbance measurement at 380nm.

### Biliverdin purification

#### Purification

Ammonium acetate (0.1M, 1.5L) was mixed with biliverdin in buffered methanol (60% 0.1 M ammonium acetate/40% methanol, vol vol^-1^, 1L) and the mixture was loaded onto a glass column (4.0mm x 300mm) packed with C18 silica beads (125Å pore, 55-105μM diameter, Waters, Manchester, UK). The column was preconditioned by sequential elution with 200mL of methanol and 200mL of buffered methanol. After loading the sample, the column was washed with 100mL buffered methanol solution. Biliverdin was eluted with 30% 0.1M ammonium acetate/70% MeOH (vol vol^-1)^ solution and collected as material in a green band. To 25mL of eluted biliverdin material was gradually added 400mL of 1mM HCl with stirring. The solution was kept at −20°C for 1h and then centrifuged for 15min at 11325xg at 4°C. The supernatant fluid was removed, the biliverdin pellet was washed and suspended in 20mL H_2_O in a 50-mL capacity plastic centrifuge tube and centrifuged for 15min at 4500xg at 4°C. The supernatant fluid was discarded, the biliverdin pellet was frozen at −80°C and then freeze-dried using a FreeZone Plus Freeze Dry System (Labconco, Kansas City, MO USA).

### Biliverdin characterization

#### Absorbance spectra

Absorbance spectra (300 and 800nm) were obtained using a SpectraMax Plus384 Absorbance Microplate Reader (Molecular Devices, Sunnyvale, CA, USA).

#### HPLC analysis

Biliverdin samples (20μL were subjected to HPLC using a Symmetry® C18 column (4.6mm x 75 mm) and a gradient of solvent A: 99.9% H_2_0, 0.1% trifluoroacetic acid and solvent B: 99.9% methanol and 0.1% trifluoroacetic acid. The elution gradient program was: 100% solvent A, 1min; 0-60% solvent B, 1min; 60-100% solvent B, 8 min, 0-100% solvent A, 1min; 100% solvent A, 4 min with a flow rate of 1mL min^-1^ using a Waters Alliance HPLC (Waters, Manchester, UK).

#### Proton NMR analysis

NMR data was collected on a JEOL Eclipse 400MhZ NMR (JEOL, Peabody, MA, USA). Biliverdin samples were dissolved in DMSO-d6 (Cambridge Isotope Labs, Andover, MA USA).

#### LC-MS analysis

Biliverdin samples were analyzed on a NanoACQUITY UPLC (Waters, Manchester, UK) and a Q-Tof Primer tandem mass spectrometer (Waters, Manchester, UK). Samples (3μL) were introduced into a Symmetry® C18 trapping column (180μM x 20mm) with NanoACQUITY Sample Manager (Waters, Manchester, UK) washed with 99% solvent A and 1% solvent B for 3min at 15μL min^-1^. Solvent A was 99.9% H_2_0, 0.1% formic acid and solvent B was 99.9% acetonitrile and 0.1% formic acid. Chemicals were eluted from the trapping column over a BEH300 C4 column with a 40min gradient (1-4% solvent B, 0.1min; 4-98% solvent B, 19.9min; 98-85% solvent B, 2min; 85% solvent B, and 10min, 85-1% solvent B, 8min) at a flow rate of 0.8μL min^-1^. MS scan time was 1.0 sec.

### NADPH biliverdin reductase activity

The enzymatic conversion of biliverdin to bilirubin was measured using the Biliverdin Reductase Assay Kit (Sigma-Aldrich, St. Louis, MO, USA). One mg of biliverdin producd by strain *E. coli* strain BL21(mHO1) was dissolved in 2mL methanol, and 0.2mL was mixed with 1mL of the kit assay buffer. The kit-supplied recombinant human biliverdin reductase A enzyme was suspended in 800μL water, and 160μL of the enzyme suspension was added to 480μL of assay buffer. Biliverdin-containing kit assay buffer (50μL), biliverdin reductase solution (200μL), and NADPH solution (0.24mg mL^-1^ NADPH in assay buffer, 750μL) were combined and the absorbance spectrum between 300-800nm was measured at 0, 15, 30, 45, 60, 90, 145, 240 and 360min using a SpectraMax Plus384 Absorbance Microplate Reader (Molecular Devices, Sunnyvale, CA, USA).

## Results and discussion

### Effect of carbon source on biliverdin production

Several potential carbon sources, alone and in combination, were examined for their abilities to support biliverdin production by *E. coli* strains BL21(HO1) and BL21(mHO1) growing in LB medium (Table 1). Lactose at 2 and 2.5% (wt vol^-1^) alone or in combination with D-glucose yielded green cultures containing 2 to 4mg L^-1^ biliverdin without IPTG addition. *E. coli* strain BL21(HO1) cultures grown with D-mannitol (alone or in combination with glucose or sorbitol) also yielded green cultures whereas other carbon compounds and combinations (Table 1) yielded brown or yellow-green and pale green cultures containing <0.2 and <2mg L^-1^ biliverdin, respectively. Similarly, *E. coli* strain BL21(mHO1) produced enhanced amounts of biliverdin with lactose alone or in combination with D-glucose. These results show that certain carbohydrates and particularly lactose (2 to 2.5% wt vol^-1^), alone or in combination with D-glucose, and D-mannitol (2 to 5% wt vol^-1^) support higher levels of biliverdin production by *E.* coli strains BL21(HO1) and BL21(mHO1) growing in LB medium as compared to other carbon sources. With lactose, the addition of IPTG was not required for enhanced biliverdin production offering a practical and economic advantage for large-scale, commercial production of biliverdin.

**Table 1:** **Biliverdin production by*E. coli*(HO1) and*E. coli*(mHO-1) growing in LB medium supplemented with various carbon sources**

***E.coli*Strain**	**Carbon Source**	**Conc. %**	**IPTG**^**a**^	**Pigment**^**b**^
BL21(HO1)	D-glucose	1	+	pale green
		2	+	pale green
		5	+	yellow green
	sucrose	1	+	brown
	D-mannitol	1	+	pale green
		2	+	pale green
		5	+	green
	D-sorbitol	1	+	yellow green
		5	+	yellow green
		10	+	yellow green
	lactose	1	-	pale green
		2.5	-	green
		5	-	yellow green
		10	-	yellow green
	D-mannitol, D-glucose	1,1	+	pale green
	sucrose, D-glucose	1,1	+	pale green
	D-mannitol, D-sorbitol	2.5, 2.5	+	green
	D-mannitol, D-sorbitol	2, 5	+	green
	D-mannitol, D-sorbitol	5, 5	+	green
	lactose, D-glucose	2, 2	-	green
	succinate	5	+	pale green
	malate	5	+	pale green
	citrate	5	+	pale green
	L- glutamate	5	+	pale green
	L- glutamate	5	+	yellow green
BL21(mHO1)	D-glucose	1	+	green
	D-mannitol	5	+	green
	lactose	2.5	-	green
	lactose, D-glucose	2, 2	-	green

^**a**^Isopropyl-β-thiogalactoside (IPTG), 0.5 mM added (+), or not added (−).

^**b**^ Biliverdin concentrations were 2 to 4mg L^-1^ (green), <2 to >0.2 mg L^-1^ (pale green) and <0.2mg L^-1^ (yellow green and brown).

### Bioreactor batch culture production of biliverdin

**Figure 3:**
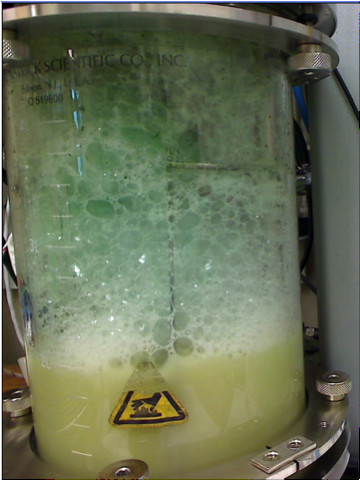
**Biliverdin production from *E. coli *strain BL21 (mHO1) growing in modified ZY medium in a New Brunswick Bioflo 310 bioreactor.** Biliverdin is subsequently extracted from the green-colored material that accumulates above the culture surface.

Based on observations that lactose enhanced biliverdin production, modified ZY medium containing 2% wt vol^-1^ lactose was used to grow *E. coli* strains BL21(HO1) and BL21(mHO1) in 2 L volumes in a New Brunswick Scientific Bioflo 310 Controller bioreactor. Consistent biliverdin production was achieved with 40% dissolved O_2_, agitation between 280 and 500 rpm, and continuous feeding of lactose (2% wt vol^-1^) and glycerol (10% wt vol^-1^) in fed-batch mode initiated 4h after culture inoculation (exponential growth phase). Biliverdin was visible as and collected in green material that accumulated in the foam above the culture liquid surface (Figure 3). Biliverdin was identified and quantitated by HPLC analyses of the collected material. *E. coli* strains BL21(HO1) and *E. coli* BL21(mHO1) produced between 2.5 and 5mg (n=3, average 3.3mg) and between 5.3 to 7.5mg (n=9, average 6.4mg) of biliverdin, respectively, per L of culture. Therefore, *E. coli* strain BL21(mHO1) produced nearly twice the amount of biliverdin than *E.coli* strain BL21(HO1) in the bioreactor cultures growing in modified ZY medium in fed-batch mode with lactose and glycerol. In contrast, the two strains produced approximately the same amounts of biliverdin when grown in LB medium with lactose (2.5% wt vol ^-1^) in small shaker flasks (Table 1). When peptone was included in the fed-batch medium (together with lactose and glycerol), *E. coli* strain BL21(mHO1) bioreactor cultures produced between18.4 to 25.3mg L^-1^ (n=11, average of 23.8 mg L^-1^) of biliverdin. *E.coli* strain BL21(HO1) produced between 3 and 3.9mg L^-1^ (n=9, average of 3.3 mgL^-1^) in modified NBS medium in batch mode.

Biliverdin production was also achieved in a 100L bioreactor (Braun UE-100D) batch mode cultures of *E. coli* strain BL21(HO1) grown in NBS medium containing 2% (wt vol^-1^) lactose. Biliverdin yields ranging between 200 to 311mg (n=5, average 212) were achieved. This rate of production was similar to that achieved by *E. coli* strain BL21(HO1) in the 2L bioreactor (Bioflo 310) batch mode cultures (average rate: 3.3mg L^-1^) indicating that biliverdin production by *E. coli* strain BL21(HO1) is scalable to larger volumes and quantities.

### HO expression and activity

**Figure 4:**
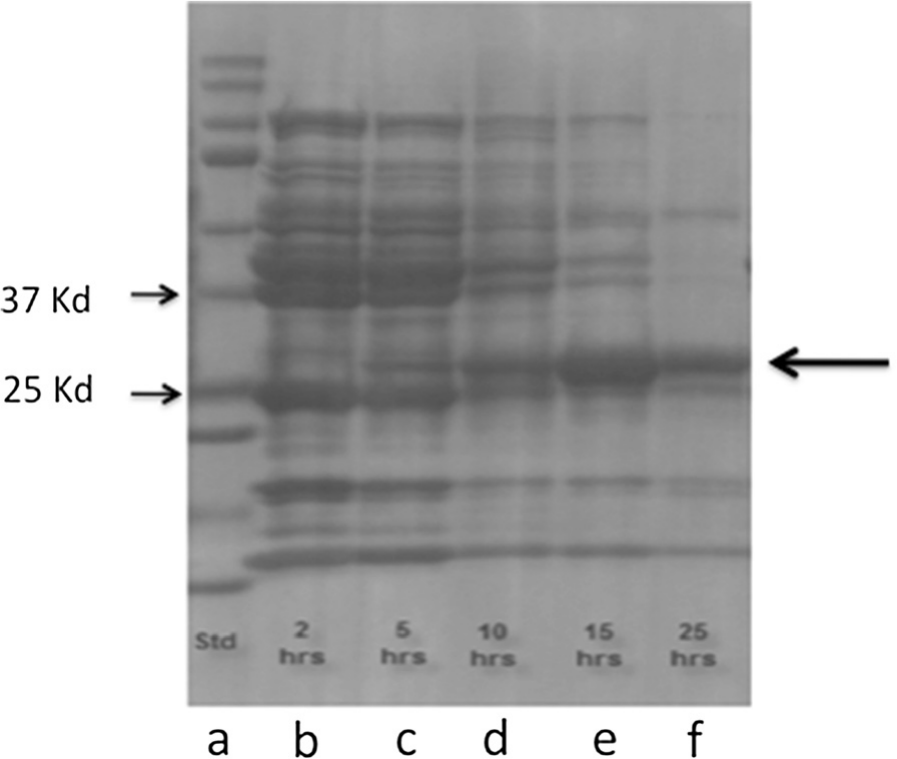
**SDS-PAGE of eluted solutions from Ni-NTA columns of cell extracts derived from bioreactor cultures of*E. coli*strain BL21 (mHO1) harvested at various times during growth on ZY medium.** Gel lanes are: mixture of protein molecular size standards (a), and cell extracts from cultures harvested at 2h, (b), 5h (c), 10h (d), 15h (e) and 25 h (f) after culture inoculation. Expression of 29Kd ho1 is evident (right arrow) and not visible when derived from cells grown without lactose or glucose + IPTG (not shown). Gel positions of 37Kd and 25Kd protein markers are indicated by arrows (left side).

When grown in 2L bioreactor cultures, *E. coli* strain BL21(mHO1) cells contained Ni-NTA recoverable proteins with molecular size ~29 Kd and detectable initially between 2 to 5h after inoculation and then until growth was terminated (25h) (Figure 4). The proteins were equivalent in size to ho1 of *Synechocystis* PCC6803 with a 6x-His tag (i.e. 28.7 Kd) and the gel excised protein showed sequence similarity to the cyanobacterial ho1 (31% sequence coverage, Mascot protein score =146). Extracts of *E. coli* strain BL21(mHO1) cells harvested at 25h of bioreactor growth had HO activities of 80 pmol hr^-1^ mg protein^-1^ whereas extracts from *E. coli* strain BL21 Star™ (DE3) cells showed no or barely detectable activities (<5 pmol hr^-1^ mg protein^-1^). These results confirmed that *E. coli* strain BL21(mHO1) synthesized an HO enzyme when grown under conditions that allowed accumulation of green pigment determined to be biliverdin (see below).

### Identification of biliverdin IXα

**Figure 5:**
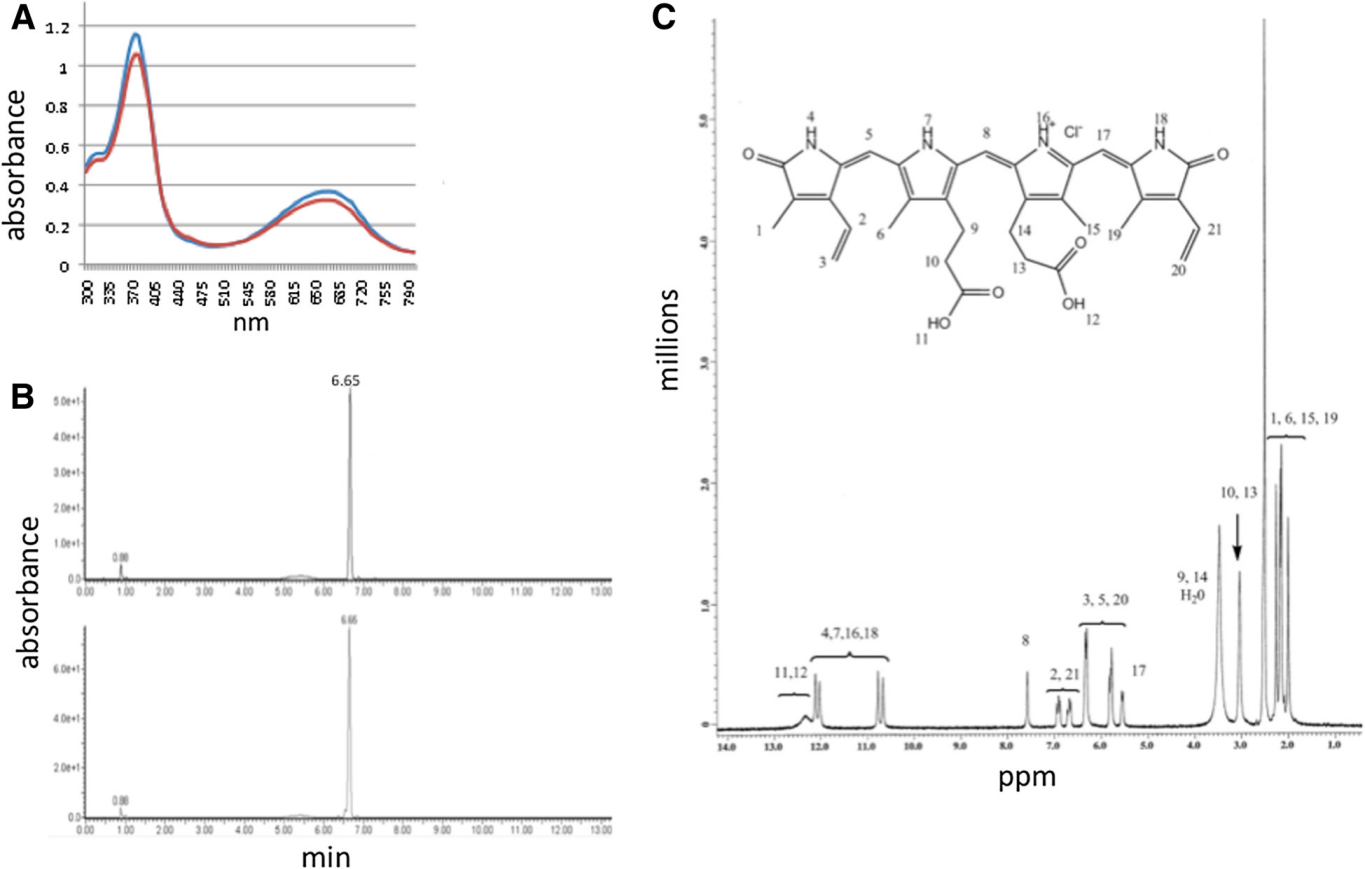
**Spectral and chromatographic analyses of biliverdin produced by bioreactor cultures of*E. coli*BL21 (mHO1).** (**A**) Absorbance spectra of biliverdin produced by *E. coli* BL21 (mHO1) (*red*) and commercial biliverdin IXα derived from an animal source (*blue*). (**B**) HPLC chromatograms of biliverdin produced by *E. coli* BL21 (mHO1) (*top*) and commercial biliverdin IXα derived from an animal source (*bottom*). (**C**) One dimensional proton NMR (400 Mhz) spectrum of *E. coli* BL21 (mHO1)-produced biliverdin in DMSO-d6. ^1^H NMR (400 MHz, DMSO) signal assignments are: 12.32 (s; 2H); 12.1(s; 1H); 12.01 (s; 1H); 10.78 (s; 1H); 10.67 (s; 1H); 7.58 (s; 1H); 6.91 (t, J= 15.6 Hz; 1H); 6.69 (t, J= 15.2 Hz; 1H); 6.32 (d, J= 12.2 Hz; 3H); 5.82 (d, J=10.8 Hz; 1H); 5.56 (d, J= 11.2 Hz; 1H); 5.78 (s; 1H); 3.05 (m; 4H); 3.43 (m; 4H); 2.18 (s; 3H); 2.27 (s; 3H); 2.15 (s; 3H); 2.01 (s; 3H). The spectrum is similar to biliverdin IXα derived from animal sources [36].

The identity of the biliverdin extracted from *E. coli* strain BL21(mHO1) cultures as biliverdin IXα was indicated by comparisons to authentic biliverdin IXα using absorbance spectroscopy, HPLC, proton NMR spectroscopy (Figure 5) and mass spectroscopy (mass 582.2). The degree of purity was >98% based on HPLC profiles (biliverdin IXα retention time of 6.6 min, Figure 5B).

### *E. coli* produced biliverdin as substrate for biliverdin reductase A

**Figure 6:**
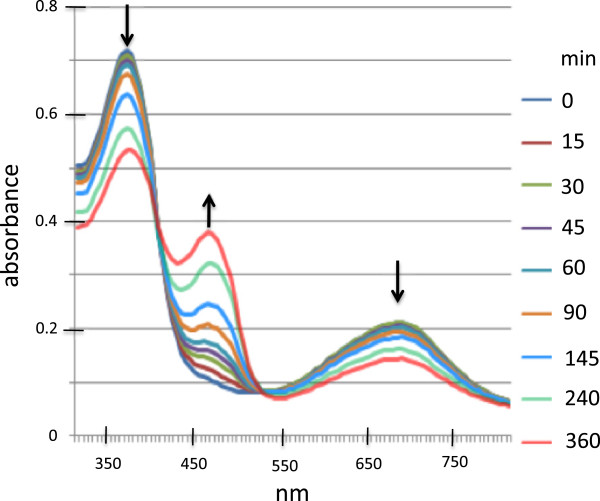
**Absorbance spectra at various times during bilirubin formation from *E. coli *BL21 (mHO1)-produced biliverdin catalyzed by human recombinant biliverdin reductase A.** NADPH-dependent reduction was monitored spectrophotometrically for 6h. The arrows indicate the direction of absorbance change over time during the reaction.

Purified biliverdin produced by *E. coli* BL21(mHO1) was reduced to bilirubin IXα by recombinant human biliverdin reductase A and NADPH (Figure 6). Since human biliverdin reductase A specifically uses biliverdin IXα as substrate [1,35], this result confirms the identity of the *E. coli* BL21(mHO1) produced biliverdin as the IXα isomer. It also suggests that the produced biliverdin has therapeutic potential because of its substrate interaction with the human enzyme and its facile conversion to bilirubin IXα.

## Conclusions

Methods for the scalable production of biliverdin by *E. coli* cultures were developed. Production is enhanced with the use of an altered version of a cyanobacterial *ho1* gene that is sequence-optimized for *E. coli* expression. The produced biliverdin is solely the physiologically relevant IXα isomer and is easily obtained at a high degree of purity (>98%). Its purity and ability to serve as substrate for human NADPH biliverdin reductase A suggest its potential as a clinically useful therapeutic for inflammatory diseases and conditions. When commercially produced for therapeutic applications, the biliverdin IXα preparations will undoubtedly require screening for and elimination of endotoxin contaminants that are a consequence and limitation of industrial scale production by *E. coli* cultures.

## Abbreviations

HO: Heme oxygenase; ROS: Reactive oxygen species; NAPDH: Reduced nicotinamide dinucleotide phosphate; IPTG: Isopropyl-β−thiogalactopyranoside; HPLC: High performance liquid chromatography; UPLC: Ultra high performance liquid chromatography; Ni-NTA: Nickel-nitriloacetic acid; NMR: Nuclear magnetic resonance.

## Competing interests

A utility patent application to the U.S. Patent and Trademark Office (No. 12/939,880, filed November 4, 2010) on topics related to the contents of this manuscript is pending. DC, JDB, YK and JYT are supported by Utah State University that has applied for the patent.

## Authors’ contributions

DC designed and conducted experiments related to gene cloning, protein expression, biliverdin purification and characterization and wrote parts of the manuscript. JDB and YK designed and conducted microbiological and bioreactor experiments. JB conducted chemical analysis and purification protocols. JYT designed experiments, coordinated the research, and wrote the manuscript. All authors read and approved the final manuscript.

## References

[B1] McDonagh AF Turning green to gold Nat Struct Biol 2001 8 3 198 200 10.1038/84915 11224558

[B2] Sedlak TW Snyder SH Bilirubin benefits: cellular protection by a biliverdin reductase antioxidant cycle Pediatrics 2004 113 6 1776 1782 10.1542/peds.113.6.1776 15173506

[B3] Soares MP Bach FH Heme oxygenase-1: from biology to therapeutic potential Trends Mol Med 2009 15 2 50 58 10.1016/j.molmed.2008.12.004 19162549

[B4] Baranano DE Rao M Ferris CD Snyder SH Biliverdin reductase: a major physiologic cytoprotectant Proc Natl Acad Sci USA 2002 99 25 16093 16098 10.1073/pnas.252626999 12456881 PMC138570

[B5] Maghzal GJ Leck MC Collinson E Li C Stocker R Limited role for the bilirubin-biliverdin redox amplification cycle in the cellular antioxidant protection by biliverdin reductase J Biol Chem 2009 284 43 29251 29259 10.1074/jbc.M109.037119 19690164 PMC2785555

[B6] McDonagh AF The biliverdin-bilirubin antioxidant cycle of cellular protection: missing a wheel? Free Rad Biol Med 2010 49 5 814 820 10.1016/j.freeradbiomed.2010.06.001 20547221

[B7] Sedlak TW Snyder SH Cycling the wagons for biliverdin reductase J Biol Chem 2009 284 46 le11 author reply le12 10.1074/jbc.L109.037119 19897493 PMC2786007

[B8] Fujii M Inoguchi T Sasaki S Maeda Y Zheng J Kobayashi K Takayanagi R Bilirubin and biliverdin protect rodents against diabetic nephropathy by downregulating NAD(P)H oxidase Kidney Int 2010 78 9 905 919 10.1038/ki.2010.265 20686447

[B9] Nakao A Murase N Ho C Toyokawa H Billiar TR Kanno S Biliverdin administration prevents the formation of intimal hyperplasia induced by vascular injury Circulation 2005 112 4 587 591 10.1161/CIRCULATIONAHA.104.509778 16027253

[B10] Nakao A Otterbein LE Overhaus M Sarady JK Tsung A Kimizuka K Nalesnik MA Kaizu T Uchiyama T Liu F Biliverdin protects the functional integrity of a transplanted syngeneic small bowel Gastroenterol 2004 127 2 595 606 10.1053/j.gastro.2004.05.059 15300591

[B11] Overhaus M Moore BA Barbato JE Behrendt FF Doering JG Bauer AJ Biliverdin protects against polymicrobial sepsis by modulating inflammatory mediators Gastrointest Liver Physiol 2006 290 4 G695 703 10.1152/ajpgi.00152.2005 16537973

[B12] Yamashita K McDaid J Ollinger R Tsui T-Y Berberat PO Usheva A Csizmadia EVA Smith RN Soares MP Bach FH Biliverdin, a natural product of heme catabolism, induces tolerance to cardiac allografts FASEB J 2004 18 6 765 767 14977878 10.1096/fj.03-0839fje

[B13] Bellner L Wolstein J Patil KA Dunn MW Laniado-Schwartzman M Biliverdin rescues the HO-2 null mouse phenotype of unresolved chronic inflammation following corneal epithelial injury Invest Ophthalmol Vis Sci 2011 52 6 3246 3253 10.1167/iovs.10-6219 21345995 PMC3109026

[B14] Wegiel B Baty CJ Gallo D Csizmadia E Scott JR Akhavan A Chin BY Kaczmarek E Alam J Bach FH Cell surface biliverdin reductase mediates biliverdin-induced anti-inflammatory effects via phosphatidylinositol 3-kinase and Akt J Biol Chem 2009 284 32 21369 21378 10.1074/jbc.M109.027433 19509285 PMC2755861

[B15] Wegiel B Gallo D Csizmadia E Roger T Kaczmarek E Harris C Zuckerbraun BS Otterbein LE Biliverdin inhibits Toll-like receptor-4 (TLR4) expression through nitric oxide-dependent nuclear translocation of biliverdin reductase Proc Natl Acad Sci 2011 108 46 18849 18854 10.1073/pnas.1108571108 22042868 PMC3219137

[B16] Florczyk UM Jozkowicz A Dulak J Biliverdin reductase: new features of an old enzyme and its potential therapeutic significance Pharmacol Rep 2008 60 1 38 48 18276984 PMC5536200

[B17] Wegiel B Otterbein L Go green: the anti-inflammatory effects of biliverdin reductase Front Pharmacol 2012 3 47 1 8 22438844 10.3389/fphar.2012.00047 PMC3306015

[B18] Wang H Ferran C Attanasio C Calise F Otterbein LE Induction of protective genes leads to islet survival and function J Transplant 2011 2011 141898 22220267 10.1155/2011/141898 PMC3246756

[B19] Fondevila C Katori M Lassman C Carmody I Busuttil RW Bach FH Kupiec-Weglinski JW Biliverdin protects rat livers from ischemia/reperfusion injury Transplant Proc 2003 35 5 1798 1799 10.1016/S0041-1345(03)00720-6 12962799

[B20] Ollinger R Bilban M Erat A Froio A McDaid J Tyagi S Csizmadia E Graca-Souza AV Liloia A Soares MP Bilirubin: A natural inhibitor of vascular smooth muscle cell proliferation Circulation 2005 112 7 1030 1039 10.1161/CIRCULATIONAHA.104.528802 16087796

[B21] Sarady-Andrews JK Liu F Gallo D Nakao A Overhaus M √ñllinger R Choi AM Otterbein LE Biliverdin administration protects against endotoxin-induced acute lung injury in rats Amer J Physiol - Lung Cell Mol Physiol 2005 289 6 L1131 L1137 10.1152/ajplung.00458.2004 16155084

[B22] Zhu Z Wilson AT Luxon BA Brown KE Mathahs MM Bandyopadhyay S McCaffrey AP Schmidt WN Biliverdin inhibits hepatitis C virus nonstructural 3/4A protease activity: mechanism for the antiviral effects of heme oxygenase? Hepatol 2010 52 6 1897 1905 10.1002/hep.23921 PMC3058505 21105106

[B23] McPhee F Caldera PS Bemis GW McDonagh AF Kuntz ID Craik CS Bile pigments as HIV-1 protease inhibitors and their effects on HIV-1 viral maturation and infectivity in vitro Biochem J 1996 320 Pt 2 681 686 8973584 10.1042/bj3200681 PMC1217983

[B24] Nakagami T Taji S Takahashi M Yamanishi K Antiviral activity of a bile pigment, biliverdin, against human herpesvirus 6 (HHV-6) in vitro Microbiol Immunol 1992 36 4 381 390 1328826 10.1111/j.1348-0421.1992.tb02037.x

[B25] Ikeda N Inoguchi T Sonoda N Fujii M Takei R Hirata E Yokomizo H Zheng J Maeda Y Kobayashi K Biliverdin protects against the deterioration of glucose tolerance in db/db mice Diabetologia 2011 54 8 2183 2191 10.1007/s00125-011-2197-2 21614569

[B26] Beale SI Cornejo J Biosynthesis of phycocyanobilin from exogenous labeled biliverdin in *Cyanidium caldarium* Arch Biochem Biophys 1983 227 1 279 286 10.1016/0003-9861(83)90372-7 6416181

[B27] Elich TD McDonagh AF Palma LA Lagarias JC Phytochrome chromophore biosynthesis. Treatment of tetrapyrrole-deficient Avena explants with natural and non-natural bilatrienes leads to formation of spectrally active holoproteins J Biol Chem 1989 264 1 183 189 2909515

[B28] Muramoto T Tsurui N Terry MJ Yokota A Kohchi T Expression and biochemical properties of a ferredoxin-dependent heme oxygenase required for phytochrome chromophore synthesis Plant Physiol 2002 130 4 1958 1966 10.1104/pp.008128 12481078 PMC166706

[B29] Rhie GE Beale SI Phycobilin biosynthesis: reductant requirements and product identification for heme oxygenase from *Cyanidium caldarium* Arch Biochem Biophys 1995 320 1 182 194 10.1006/abbi.1995.1358 7793979

[B30] Wilks A Schmitt MP Expression and characterization of a heme oxygenase (Hmu O) from *Corynebacterium diphtheriae* Iron acquisition requires oxidative cleavage of the heme macrocycle. J Biol Chem 1998 273 2 837 841 10.1074/jbc.273.2.837 9422739

[B31] Schluchter WM Glazer AN Characterization of cyanobacterial biliverdin reductase Conversion of biliverdin to bilirubin is important for normal phycobiliprotein biosynthesis. J Biol Chem 1997 272 21 13562 13569 10.1074/jbc.272.21.13562 9153203

[B32] Giraud E Fardoux J Fourrier N Hannibal L Genty B Bouyer P Dreyfus B Vermeglio A Bacteriophytochrome controls photosystem synthesis in anoxygenic bacteria Nat 2002 417 6885 202 205 10.1038/417202a 12000965

[B33] Kaneko T Sato S Kotani H Tanaka A Asamizu E Nakamura Y Miyajima N Hirosawa M Sugiura M Sasamoto S Kimura T Hosouchi T Matsuno A Muraki A Nakazaki N Naruo K Okumura S Shimpo S Takeuchi C Wada T Watanabe A Yamada M Yasuda M Tabata S Sequence analysis of the genome of the unicellular cyanobacterium Synechocystis sp. strain PCC6803. II. Sequence determination of the entire genome and assignment of potential protein-coding regions DNA Res 1996 3 3 109 136 10.1093/dnares/3.3.109 8905231

[B34] Richaud C Zabulon G The heme oxygenase gene (pbsA) in the red alga *Rhodella violacea* is discontinuous and transcriptionally activated during iron limitation Proc Natl Acad Sci USA 1997 94 21 11736 11741 10.1073/pnas.94.21.11736 9326680 PMC23621

[B35] McDonagh AF Palma LA Preparation and properties of crystalline biliverdin IX alpha. Simple methods for preparing isomerically homogeneous biliverdin and [14C[biliverdin by using 2,3-dichloro-5,6-dicyanobenzoquinone Biochem J 1980 189 2 193 208 7458909 10.1042/bj1890193 PMC1161990

[B36] McDonagh AF Biliverdin, immune-mediated liver injury, and the Gigo effect Hepatol 2005 41 3 680 681 author reply 681 10.1002/hep.20587 15723311

[B37] Ishikawa K Sato M Yoshida T Expression of rat heme oxygenase in *Escherichia coli* as a catalytically active, full-length form that binds to bacterial membranes Eur J Biochem 1991 202 1 161 165 10.1111/j.1432-1033.1991.tb16357.x 1935972

[B38] Wilks A Ortiz de Montellano PR Rat liver heme oxygenase. High level expression of a truncated soluble form and nature of the meso-hydroxylating species J Biol Chem 1993 268 30 22357 22362 8226746

[B39] Cornejo J Willows RD Beale SI Phytobilin biosynthesis: cloning and expression of a gene encoding soluble ferredoxin-dependent heme oxygenase from Synechocystis sp. PCC 6803 Plant J 1998 15 1 99 107 10.1046/j.1365-313X.1998.00186.x 9744099

[B40] Pendrak ML Roberts DD Methods for the production of biliverdin US 2005/0209305 A1 2005 USA: US Patent Application Publication

[B41] Ding ZK Xu YQ Purification and characterization of biliverdin IXα from Atlantic salmon (*Salmo salar*) bile Biochem (Moscow) 2002 67 927 932 10.1023/A:1019974822667 12223093

[B42] Sambrook J Fritsch EF Maniatis T Molecular cloning: a laboratory manual 1989 Cold Spring Harbor, New York: Cold Spring Harbor Laboratory

[B43] Vyas R Basics of benchtop fermentor operation for growth of E. coli BioTechniques Protocol Guide 2008 2008:29. Print

[B44] Studier FW Protein production by auto-induction in high density shaking cultures Protein Expr Purif 2005 41 1 207 234 10.1016/j.pep.2005.01.016 15915565

[B45] Tenhunen R Marver HS Schmid R The enzymatic conversion of heme to bilirubin by microsomal heme oxygenase Proc Natl Acad Sci USA 1968 61 2 748 755 10.1073/pnas.61.2.748 4386763 PMC225223

[B46] O'Carra P Colleran E Separation and identification of biliverdin isomers and isomer analysis of phycobilins and bilirubin J Chromatogr 1970 50 3 458 468 5449461 10.1016/s0021-9673(00)97973-1

